# Depressed Hypoxic and Hypercapnic Ventilatory Responses at Early Stage of Lethal Avian Influenza A Virus Infection in Mice

**DOI:** 10.1371/journal.pone.0147522

**Published:** 2016-01-25

**Authors:** Jianguo Zhuang, Peng Gao, Zemmie Pollock, Kevin S. Harrod, Fadi Xu

**Affiliations:** Pathophysiology Program, Lovelace Respiratory Research Institute, 2425 Ridgecrest Drive SE, Albuquerque, NM, 87108, United States of America; Washington University School of Medicine, UNITED STATES

## Abstract

H5N1 virus infection results in ~60% mortality in patients primarily due to respiratory failure, but the underlying causes of mortality are unclear. The goal of this study is to reveal respiratory disorders occurring at the early stage of infection that may be responsible for subsequent respiratory failure and death. BALB/c mice were intranasally infected with one of two H5N1 virus strains: HK483 (lethal) or HK486 (non-lethal) virus. Pulmonary ventilation and the responses to hypoxia (HVR; 7% O_2_ for 3 min) and hypercapnia (HCVR; 7% CO_2_ for 5 min) were measured daily at 2 days prior and 1, 2, and 3 days postinfection (dpi) and compared to mortality typically by 8 dpi. At 1, 2, and 3 dpi, immunoreactivities (IR) of substance P (SP-IR) in the nodose ganglion or tyrosine hydroxylase (TH-IR) in the carotid body coupled with the nucleoprotein of influenza A (NP-IR) was examined in some mice, while arterial blood was collected in others. Our results showed that at 2 and 3 dpi: 1) both viral infections failed to alter body temperature and weight, ${\dot{\text{V}}}_{{\text{CO}}_{2}}$, or induce viremia while producing similarly high lung viral titers; 2) HK483, but not HK486, virus induced tachypnea and depressed HVR and HCVR without changes in arterial blood pH and gases; and 3) only HK483 virus led to NP-IR in vagal SP-IR neurons, but not in the carotid body, and increased density of vagal SP-IR neurons. In addition, all HK483, rather than HK486, mice died at 6 to 8 dpi and the earlier death was correlated with more severe depression of HVR and HCVR. Our data suggest that tachypnea and depressed HVR/HCVR occur at the early stage of lethal H5N1 viral infection associated with viral replication and increased SP-IR density in vagal neurons, which may contribute to the respiratory failure and death.

## Introduction

Patients infected by highly pathogenic avian influenza (HPAI) H5N1 viruses have a spectrum of clinical abnormalities, ranging from mild symptoms to respiratory failure and death [1,2]. In all fatal cases (60% mortality), patients present with cough, dyspnea, pulmonary inflammation and infiltration during the first 6 days postinfection (dpi) and subsequently develop respiratory failure (hypoxemia), leading to death several days later [1,3–6]. The current unavailability of effective vaccine coupled with the continuous zoonotic transmission of endemic H5N1 viruses in domesticated birds makes widespread epidemics highly plausible, particularly if viral mutations lead to more sustainable human-to-human transmission. Thus, both determinations of the lethality of the infection at the early stage and development of corresponding effective treatments to prevent the subsequent respiratory failure are urgently needed.

Previous studies have shown that two virus isolates from human cases in an H5N1 outbreak in 1997 have dissimilar outcomes in experimental murine infection, despite nearly identical genetic compositions. BALB/c mice infected with lethal and non-lethal H5N1 viruses isolated from patients have shown similar viral titers in lungs and pulmonary inflammation; however, only the former is neurotropic and lethal, inducing death 6–8 dpi in mice [7,8], ferrets [9], and cats [10]. Though respiratory failure is the major causative factor of H5N1 viral infection leading to death in the clinical setting, the respiratory pathophysiology at the early stage of the infection is unexplored. It is generally accepted that hypoxic and hypercapnic ventilatory responses (HVR and HCVR) are crucial in maintaining life in mammals. Their depression or deficit is responsible for the respiratory failure in many fatal diseases, such as SIDS [11–13], COPD [14,15], congenital central hypoventilation syndrome [16,17], and Leigh syndrome [18]. Therefore, we hypothesized that depressed HVR and HCVR would occur in the early stage of lethal H5N1 viral infection and could be correlated with the death.

Our study has shown HVR and HCVR depression at the early stage in lethal rather than non-lethal H5N1 viral infected mice, raising a fundamental question as to how the lethal H5N1 virus induces these disorders. It is established that HVR is mediated predominantly by stimulating carotid chemoreceptors [19]. Moreover, the evidence is accumulating that activation of bronchopulmonary C-fibers (PCFs) could abolish HVR [20,21] and CO_2_ chemoreception of the retrotrapezoid nucleus [22], a key medullary region in generating HCVR [23]. PCFs exert these modulatory impacts likely via their projections to the commissural subnucleus of the nucleus tractus solitarius (comNTS), where neurons not only receive inputs from the carotid body [24,25] but also project to neurons in the retrotrapezoid nucleus [22]. Over-expression of C-fibers and vagal (PCF) sensitization [14,26,27] depresses HVR and/or HCVR. Thus, we further hypothesized that lethal H5N1 virus was able to infect both the carotid body and PCFs, which may contribute to the development of HVR and HCVR depression.

## Materials and Methods

### Animals

The present study was approved by the Institutional Animal Care and Use Committee (IACUC) and Institutional Biosafety Committee of the Lovelace Respiratory Research Institute. All facilities were accredited by the Association for Assessment and Accreditation of Laboratory Animal Care (AAALAC) International. BALB/c female mice experiments were conducted in the Animal Biosafety Level 3 enhanced (ABSL-3+) facility. Guidelines for mice housing, environment, and comfort described in the Guide for the Care and Use of Laboratory Animals (7^th^ Edition, National Research Council) were strictly followed. Euthanasia was performed under the guidance of the American Veterinary Medical Associations (AVMA) Guidelines on Euthanasia.

### Mice handling/care and identification

Eighty pathogen-free mice were purchased from Charles River Laboratories, Inc. (Wilmington, MA) and quarantined for 2 weeks before the experiments. Mice had access to food and water ad libitum. They were 4–5 weeks of age at the day of infection, which is similar to our previous studies [7]. Temperature and humidity ranged from 16 to 22°C and 30 to 65%, respectively, and the light cycle was 12 hr on and 12 hr off. Ventilation in the study room was 0.15 air exchanges per hour. All mice handlers were vaccinated for circulating seasonal influenza strains and were not permitted to enter mice quarters if they exhibited any symptoms of upper or lower respiratory infection. Mice were identified by an IPTT-300 implantable programmable temperature and identification transponder (Bio Medic Data Systems, Inc, BMDS, Seaford, Delaware). These chips also provided subcutaneous body temperature data using a BMDS electronic proximity reader wand (WRS-6007, BMDS). The mice subjected to the survival study were monitored twice daily in the morning and in the evening. Their behavior and health conditions were checked. Their body weights and body temperatures were measured. The humane endpoints were set as body weight lose > 30% or body temperature drops to < 32 degrees or appeared moribund. Animals met the criteria were humanely sacrificed via injection of Euthasol (200 mg kg^-1^, ip). No analgesics and anesthetics were used.

### Viruses and cells

Avian influenza A H5N1 viruses were obtained from the Centers for Disease Control and Prevention (CDC) (Atlanta, GA). A/Hong Kong/483/97 (HK483) was isolated from a pharyngeal swab from a five-year-old female patient who died of the disease in the Hong Kong outbreak in 1997, while A/Hong Kong/486/97 (HK486) came from a 13-year-old female patient who was discharged from the hospital during that event [28]. All manipulations with these viruses and use of viral infected mice were conducted in the ABSL-3+ at the Lovelace Respiratory Research Institute. These viruses were propagated from the CDC stock in eggs twice to produce working stocks, aliquoted, titrated by plaque assay on Madin-Darby Canine Kidney (MDCK) cells, and stored at -80°C.

### Viral infection of mice and clinic observation

After anesthesia with isoflurane, 50 μl of vehicle, HK483 or HK486 virus (100 PFU) was intranasally given via dropwise application to the nares as previously reported [7]. The mice were closely monitored until recovery of normal postural reflexes and placed separately in their home cage. They were divided into three groups: Ctrl, HK486 and HK483. Clinical observations were conducted twice daily during -2 to 3 dpi and included temperature readings from the BMDS microchip and recording of the onset, severity, and duration of all visible changes such as abnormal respiration (cough and sneezing), excretions, behavioral characteristics, and neurological signs (i.e., paresis, torticollis, seizures, and paralysis). Mortality was counted over 8 dpi.

### Animal habituation

Mice were habituated to handling and two types of chambers. They were individually placed in a 60 ml syringe chamber (with the plunger removed) for 10 min and then moved into a whole-body unrestrained plethysmograph chamber (PLY3211, Buxco Electronics Inc., Troy, NY) with a bias flow (0.5 L min^-1^) for ~45 min. The same habituation was applied once a day for three continuous days.

### Measurements of metabolism and ${\dot{\text{V}}}_{\text{M}}$

After habituation to both chambers, ${\dot{V}}_{{CO}_{2}}$ was first measured. As reported before [29], the individual mouse was placed in the syringe chamber with an open end that was closed by a plug with an inlet connected to a flow regulator (Bias flow regulator, Buxco Research Systems, Wilmington, NC). CO_2_ concentration (by using a CO_2_ analyzer, Hewlett Packard 78356A), temperature, and humidity in the air out of the syringe were continuously measured to determine ${\dot{V}}_{{CO}_{2}}$. The animal was then placed in the plethysmograph chamber that was continuously flushed with normoxic (21% O_2_ and 79% N_2_) gas mixtures at 0.5 L min^-1^. The temperature inside the chamber was maintained at ~30.0°C as reported before [30,31] through adjusting a heating lamp outside of the chamber, by which the animal body temperature was maintained at ~36.5°C. Calibrations for flow rate and gas concentrations were made before and after each experiment. All studies were performed during 9:00 and 17:00 hours to avoid any influence from the circadian rhythm.

### Blood sample collections and measurements of pH and blood gases

Some mice were anesthetized with urethane (1200 mg kg^-1^, ip). As needed, supplemental urethane (300 mg kg^-1^, ip) was administered to completely eliminate eye-blink and limb-withdrawal reflex. The right femoral artery was isolated and cannulated, and arterial blood was sampled (100 μl) for measurements of baseline pH and blood gases using a blood gases analyzer (GEM Premier 3000, Instrumentation Lab., Lexington, MA).

### Detection of viremia with real-time quantitative PCR

After the anesthesia, the animal was subsequently euthanized (Euthasol 150 mg kg^-1^, i.p.). The blood sample was centrifuged (13,000 rpm, 4°C for 5 min) and serum was collected and placed in a -80°C freezer. Viral RNA was extracted using the Qiagen QIAamp Viral RNA Mini Kit (cat No: 52904) and was performed by H5N1-specific (NP gene) real-time quantitative PCR. PCR standards of RNA genomic copy numbers were used to determine the gene copy numbers of serum samples. All assays were repeated in triplicate on the ABI 7300 real-time PCR system (Applied Biosystems, Life Technologies Corp., Carlsbad, CA). The quantitative PCR results were analyzed with the software provided.

### Assay for viral titers in the lungs

Plaque assay was performed in MDCK cells to quantify the multiplicity of viral infection in the lungs. In brief, after euthanasia, the lungs from each mouse were harvested and homogenized in 1.0 ml of PBS with one 5 mm stainless steel bead and homogenized with a Qiagen TissueLyser (Qiagen Inc., Valencia, CA) for 2 min at 30 Hz/sec. Homogenized material was spinned down and 100 μl supernatant with ten-fold series dilution was applied to preseeded 12-well plates of MDCK cells (95% confluent) and then overlaid with agar containing 3 μg/ml of trypsin (Sigma–Aldrich, St. Louis, MO). Three days later, the plaque-forming unit (PFU) was counted after fixation, removal of the agar and staining with 1.6% w/v crystal violet.

### Immunofluorescence

Formalin-fixed and paraffin-embedded tissues of the carotid body and nodose ganglion were sectioned (10 μm thick), deparaffinized, rehydrated, washed, and permeabilized and non-specific sites were blocked for immunofluorescence. The sections were incubated with the solution containing the primary antibody or antibodies mixture to specifically target the nucleoprotein of H5N1 influenza A (NP, mouse anti-influenza A monoclonal IgG 1:1000, EMD Millipore, Billerica, MA), substance P (SP, guinea-pig anti-substance P polyclonal IgG 1:1000, EMD Millipore) in the nodose ganglion or tyrosine hydroxylase (TH, rabbit anti-tyrosine hydroxylase polyclonal IgG 1:1000, EMD Millipore) in the carotid body respectively. The antibody concentrations and incubation durations were determined and optimized prior to the experiment. The secondary antibodies were raised in goat and conjugated with Alexa Fluor 350 (blue), 488 (green), and 594 (red) alone or in combination (1:200, Life Technologies). For multilabeling, antibodies raised from different animal species were selected. The immunoreactivity was visualized using an epifluorescent microscope (Axioskop FS 2 plus, Zeiss, Germany) equipped with a CCD digital camera (Zeiss Axiocam HRm, Zeiss) and images were captured using Axiovision 4 software (Zeiss).

### Experimental protocols

Three *Study Series* were performed in this study.

*Series I* was designed to determine the effects of H5N1 viral infection at the early stage on body weight, body temperature, metabolism (${\dot{V}}_{{CO}_{2}}$), ventilation (HVR and HCVR) and the correlation between the depression of HVR/HCVR and the death date. After measuring baseline ${\dot{V}}_{{CO}_{2}}$, Ctrl (vehicle), HK483, and HK486 mice (n = 6, 15, and 7 respectively) were individually placed in the plethysmograph at -2, -1, 1, 2, and 3 dpi to record baseline ${\dot{V}}_{M}$ and baseline respiratory intervals (RR). The Ctrl, HK483 and HK486 mice came from two sets of experiments (n = 3, 7, and 4 in the first set and 3, 8, 3 in the second set). The RR is a more sensitive index than respiratory frequency to reflect a change of respiratory control [32]. We applied the Poincare analysis in which the duration of each breath was plotted *versus* the duration of the next breath as previously reported [27]. The width of the variation was calculated perpendicularly to (SD1) and along the line of identification (SD2). The ventilatory responses to hypoxia (7% O_2_ balance with N_2_ for 3 min) and hypercapnia (7% CO_2_ + 40% O_2_ balance with N_2_ for 5 min) were measured subsequently at these days. The mice were observed twice daily up to 8 or 9 dpi to define the mortality and death date.

*Series II* was conducted to collect arterial blood for testing the viral effect on blood gases/pH and venous blood and the lungs for detecting viral titers in the three groups of mice. Mice (n = 7 in each group, 4 and 3 for the first and second set, respectively) were anesthetized to collect arterial blood samples from femoral artery at 3 dpi, and euthanized for harvesting the lungs and determine the viral replication.

*Series III* was performed to identify if HK483 or HK486 viruses were able to replicate in vagal sensory neurons and the carotid body at the early stage of infection. Additional Ctrl, HK483 and HK486 mice (in the second set) were euthanized at 1 (n = 5/per group), 2 (n = 3/per group), and 3 (n = 2/per group) dpi. Following fixation via intracardiac perfusion of 4% paraformaldehyde in PBS, the nodose ganglia and the carotid body were extracted and prepared for immunofluorescence. Immunoreactivities (IR) of SP (SP-IR) and TH (TH-IR) were utilized to mark vagal sensory neurons in the nodose ganglia and glomus cells in the carotid body, respectively, while co-staining with NP-IR was employed to detect viral replication.

### Data acquisition and statistical analysis

Raw data of the airflow and CO_2_ concentrations were digitized, monitored, and recorded by PowerLab/8sp (model ML 785; ADInstruments Inc., Colorado Springs, CO) and a computer with the LabChart Pro 7 software. Respiratory variables including V_T_, f_R_, and ${\dot{\text{V}}}_{\text{M}}$; breathing variations (RR) were derived by the online calculations of the airflow signals. Body temperatures, body weight, arterial blood pH and gases were measured. All variables were expressed as absolute values with the exception that HVR and HCVR were presented as Δ% change from the baseline values. The baseline values were determined by measuring the variables for 1 min immediately before hypoxia or hypercapnia. HVR and HCVR were measured at the peak ${\dot{V}}_{M}$ response. Owing to the similarity of the results obtained from the first and second set of the experiments, the data from the two sets were grouped together for statistical analysis. Group data were reported as means ± SE. Correlations between HVR and HCVR in -2 to 3 dpi and between the depression of HVR and HCVR and the death date in HK483 mice were analyzed by using Pearson’s linear correlation analysis. Two-way ANOVA with repeated measures was used to analyze the significant differences among the three groups. If an overall test was significant, Tukey’s test was utilized for specific comparisons between individual groups. Comparisons of mortality among the three groups were performed with the Fisher exact probability test followed by multiple comparisons using Bonferroni’s test. P-values < 0.05 were considered significant.

## Results

### HK483 and HK486 viral infection have little effect on behaviors at the early stage of infection

**Fig 1:**
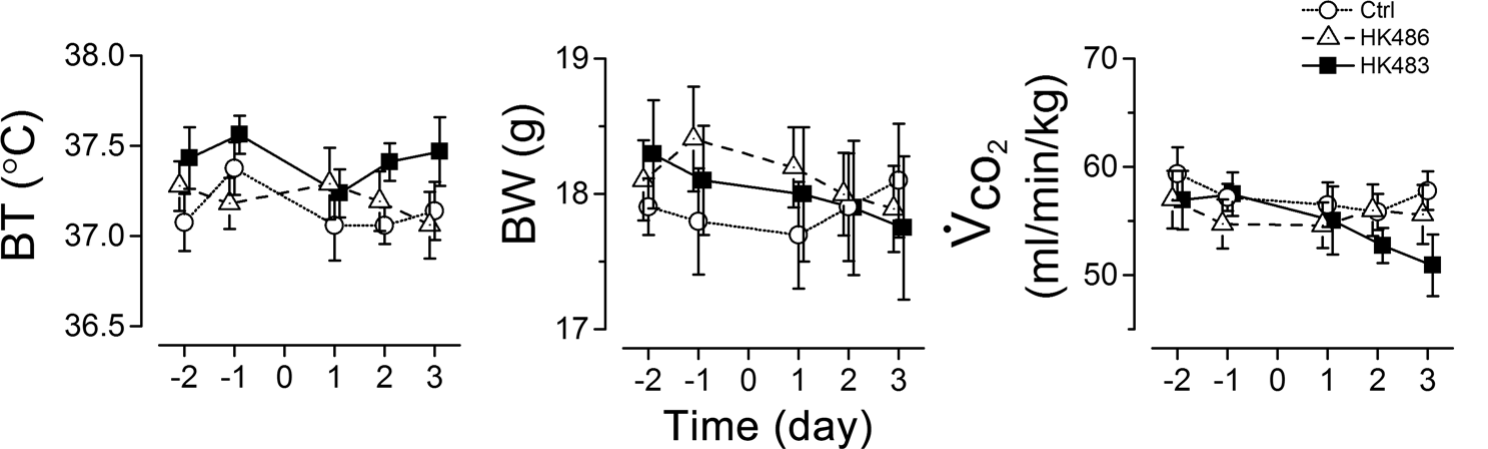
Effects of H5N1 viral infection on animal body temperature (BT), body weight (BW), and metabolism (${\dot{\text{V}}}_{{\text{CO}}_{2}}$). In each mouse of the three groups (n = 6, 15, and 7 for Ctrl, HK483 and HK486 mice), the same measures (BT, BW, ${\dot{\text{V}}}_{{\text{CO}}_{2}}$) were repeated at 2 and 1 days before and 1, 2, and 3 days after intranasal inoculation (Day -2, -1, 1, 2, and 3). Mean ± SE. As the results show, there is no significant difference of BT, BW and ${\dot{\text{V}}}_{{\text{CO}}_{2}}$ among the three groups.

At the early stage of infection, the mice infected by HK483 or HK486 showed no discernible behavior abnormalities, such as agitation and loss of appetite as compared to uninfected mice. We compared body temperature, body weight, and ${\dot{\text{V}}}_{{\text{CO}}_{2}}$ from -2 to 3 dpi and found no significant differences in these parameters among the three groups (Fig 1).

### HK483 but not HK486 virus induces tachypnea and reduces the RR variation

**Fig 2:**
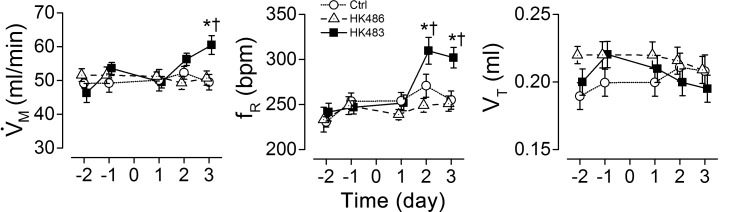
Comparison of viral infection effects on baseline ${\dot{V}}_{M}$ among Ctrl, HK483, and HK486 mice. ${\dot{\text{V}}}_{\text{M}}$ was daily measured in the individual mouse at 2 and 1 days before and 1, 2, and 3 days after intranasal inoculation (Day -2, -1, 1, 2, and 3). ${\dot{\text{V}}}_{\text{M}}$, minute ventilation; f_R_, respiratory frequency; and V_T_, tidal volume. Mean ± SE. n = 6, 15, and 7 for Ctrl, HK483 and HK486 mice. * P < 0.05 compared to the data obtained in previous day(s) and † P < 0.05 compared to Ctrl and HK486 mice at the same time-point. Tachypnea (elevation of minute ventilation) is induced in HK483 but not HK486 mice at 2 and 3 dpi.

**Fig 3:**
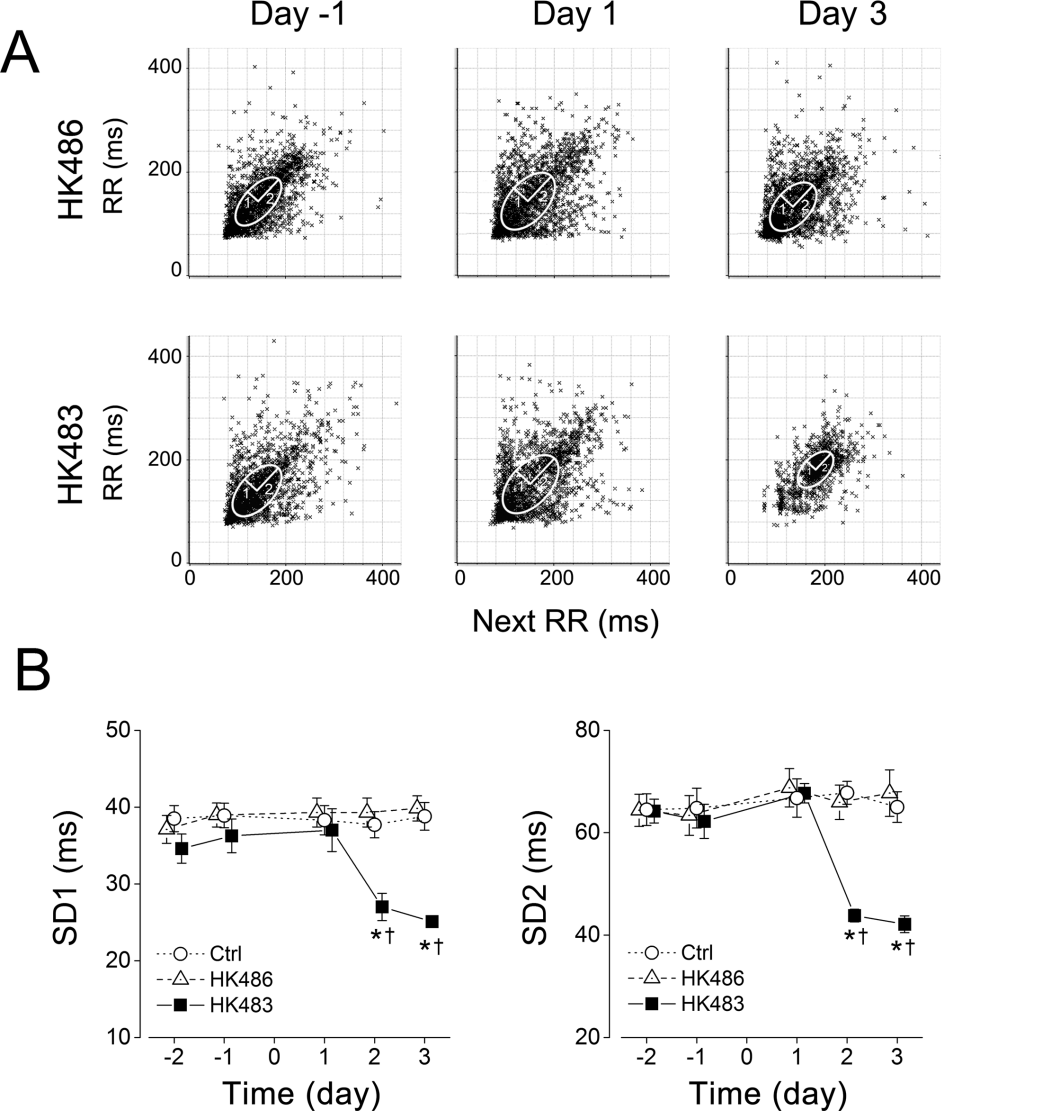
Effects of H5N1 viral infection on the variation of the baseline respiratory intervals (RR). RR was daily measured in the individual Ctrl, HK483 and HK486 mouse at 2 and 1 days before and 1, 2, and 3 days after intranasal inoculation (Day -2, -1, 1, 2, and 3). A: Poincare plots of RR in an HK486 and an HK483 mice before inoculation (Day -1) and at 1 and 3 days after intranasal inoculation (Day 1, 3). The area of the ellipse describes the distribution of the points with the width of the variation perpendicular to (SD1) and along the line of identify (SD2) [27]. B: Group data of the variability of baseline respiratory intervals (2500 intervals) in each group. Mean ± SE. n = 6, 15, and 7 for Ctrl, HK483 and HK486 mice. * P < 0.01 compared to the previous time-points; † P < 0.01, compared to Ctrl and HK486 at the given day. A less RR variation is observed after HK483 but not HK486 viral infection at 2 and 3 dpi.

We examined eupneic breathing and RR variation at -2 to 3 dpi among the mice inoculated with vehicle (Ctrl), HK483 or HK486 virus. The eupneic breathing and RR in HK486 mice were similar to those observed in Ctrl mice at -2 to 3 dpi and no changes were found in these variables until 2 dpi in HK483 mice (Figs 2 and 3). At 2 and 3 dpi, HK483 mice, compared to Ctrl and HK486 mice, presented a unique tachypnea with f_R_ elevated by ~17% (Fig 2) and significant diminution of the variation of respiratory cycle including SD1 and SD2 (Fig 3).

### HK483 virus uniquely induces depressed HVR and HCVR that correlate to the death date

**Fig 4:**
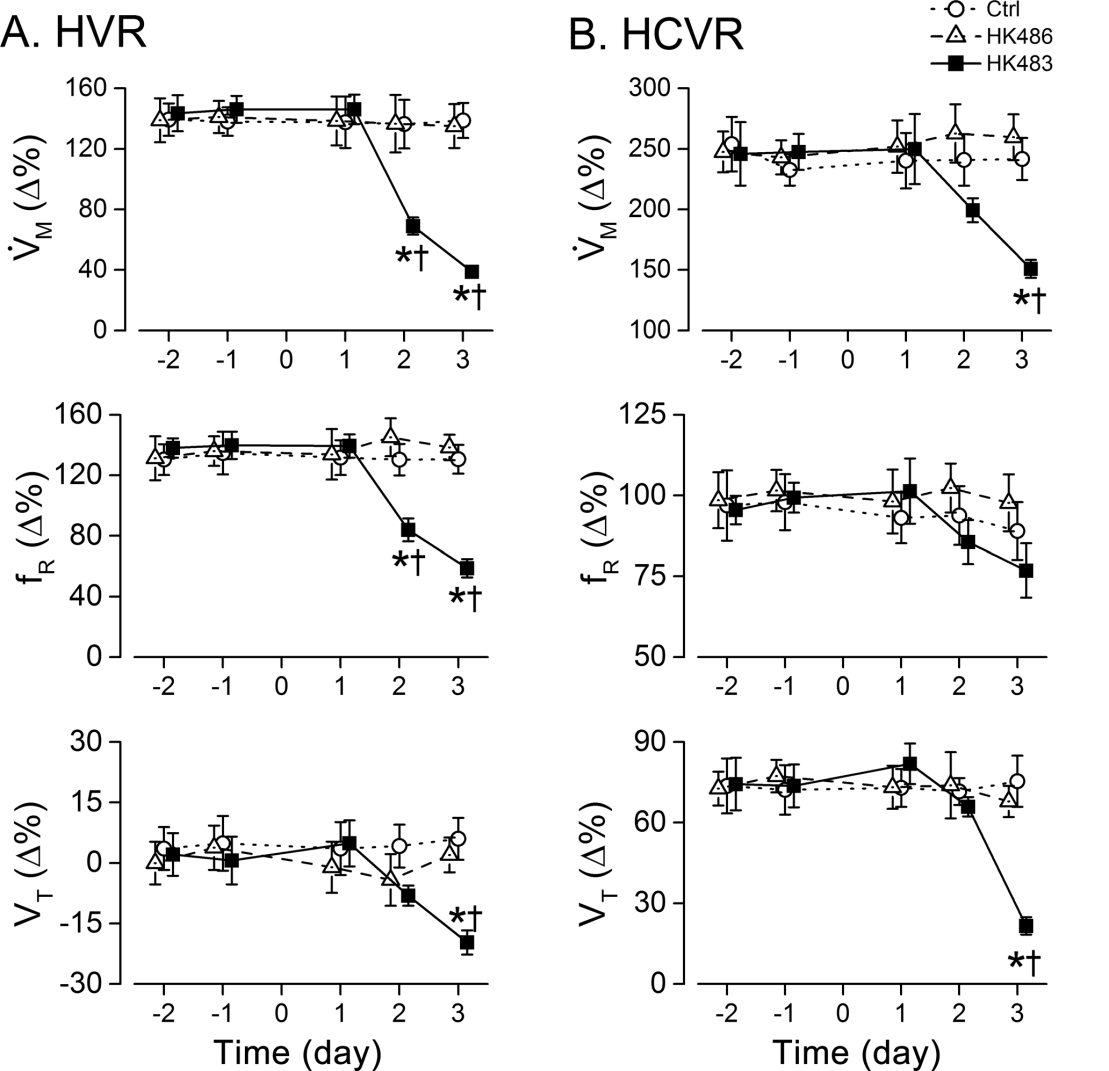
Comparison of viral infection effects on HVR (A) and HCVR (B) among Ctrl, HK483, and HK486 mice. HVR and HCVR were measured daily in individual mouse at 2 and 1 days before and 1, 2, and 3 days after intranasal inoculation (Day -2, -1, 1, 2, and 3). ${\dot{\text{V}}}_{\text{M}}$, minute ventilation; f_R_, respiratory frequency; and V_T_, tidal volume. Mean ± SE. n = 6, 15, and 7 for Ctrl, HK483 and HK486 mice. * P < 0.05 compared to the data obtained in previous day(s) and † P < 0.05 compared to Ctrl and HK486 mice at the same time-point. HK483 rather than HK486 viral infection produces HVR and HCVR depression at 2 and 3 dpi.

**Fig 5:**
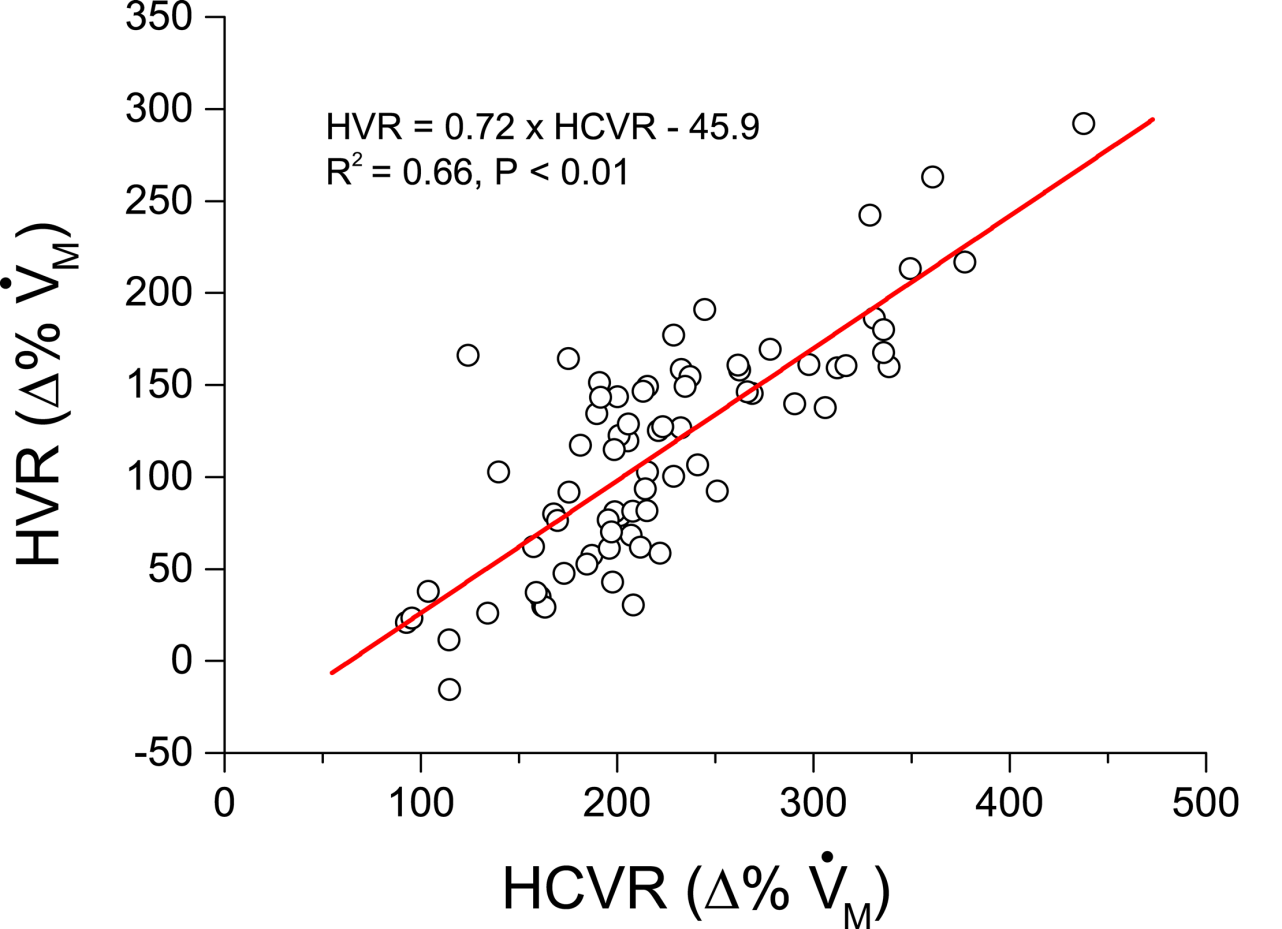
Correlation of HVR and HCVR values (${\dot{\text{V}}}_{\text{M}}$) in HK483 mice. The values of HRV measured at -2, -1, 1, 2, and 3 days after intranasal inoculation were plotted against those of HCVR at the same times. n = 15 HK483 mice for each day (total 5 days, 75 data points). The significant correlation suggests that a poor HVR is often associated with an inadequate HCVR in HK483 mice.

**Fig 6:**
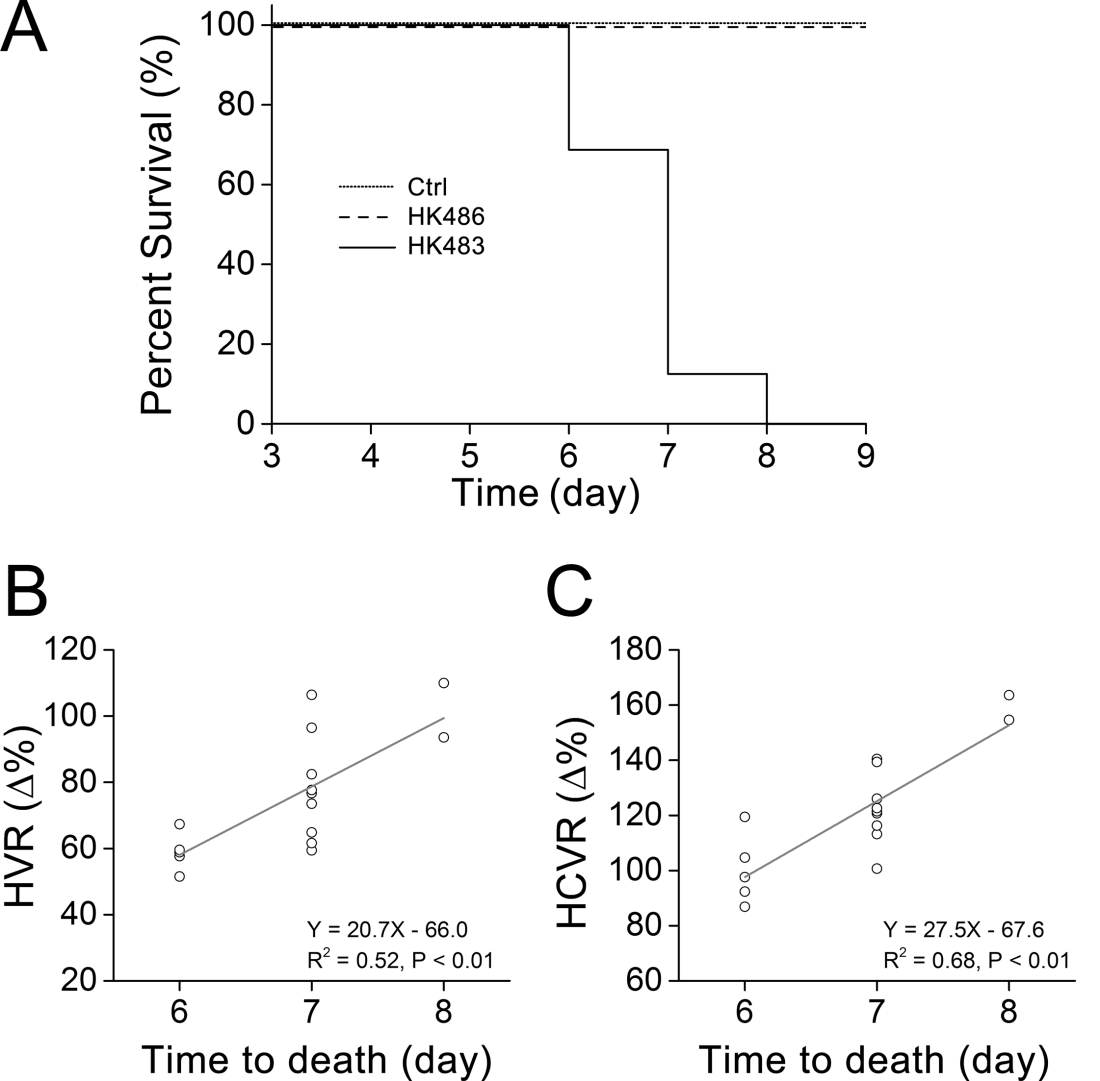
The time to death after viral infection and its correlation with the severity of HVR/HCVR depression. A: Mortality of Ctrl, HK483, and HK486 mice over the infection period (up to 9 dpi). B and C: HVR and HCVR of individual HK483 mice were plotted against their corresponding death day (at 6, 7, and 8 dpi). Mean ± SE. A: n = 6, 15, and 7 for Ctrl, HK483 and HK486 mice, respectively. B and C: n = 15 HK483 mice. HK483 but not HK486 viral infection led to death at 6–8 dpi and the mice with the larger decrement in HVR or HCVR died earlier.

With respect to HVR and HCVR, HK483, but not HK486, infection led to a striking depression of both HVR (50% and 70%↓ for 2 and 3 dpi) and HCVR (20% and 40%↓ for 2 and 3 dpi) (Fig 4). Depressed HVR was mainly the result of lowered f_R_ response with little change in V_T_ response, while depressed HCVR was exclusively associated with a distinct reduction in V_T_ response. All of the responses (HVR and HCVR) disappeared within 3 min after cession of the challenge similarly in all mice tested. No coughing and sneezing were observed throughout the experiment in all of the mice tested. The decrease in HVR caused by the viral infection was accompanied by a corresponding decrease in HCVR in HK483 mice as shown by the close correlation between the HVR and HCVR values over the experiment days (Fig 5). All HK483 mice, but none of the Ctrl and HK486 mice, died at 6–8 dpi (Fig 6A). The degrees of HVR and HCVR depression were significantly correlated with the time to death, i.e., the mice with the larger decrement in HVR or HCVR died earlier (Fig 6B and 6C).

### HK483 and HK486 viruses fail to alter arterial blood gases/pH and produce similar lung virus titer

**Fig 7:**
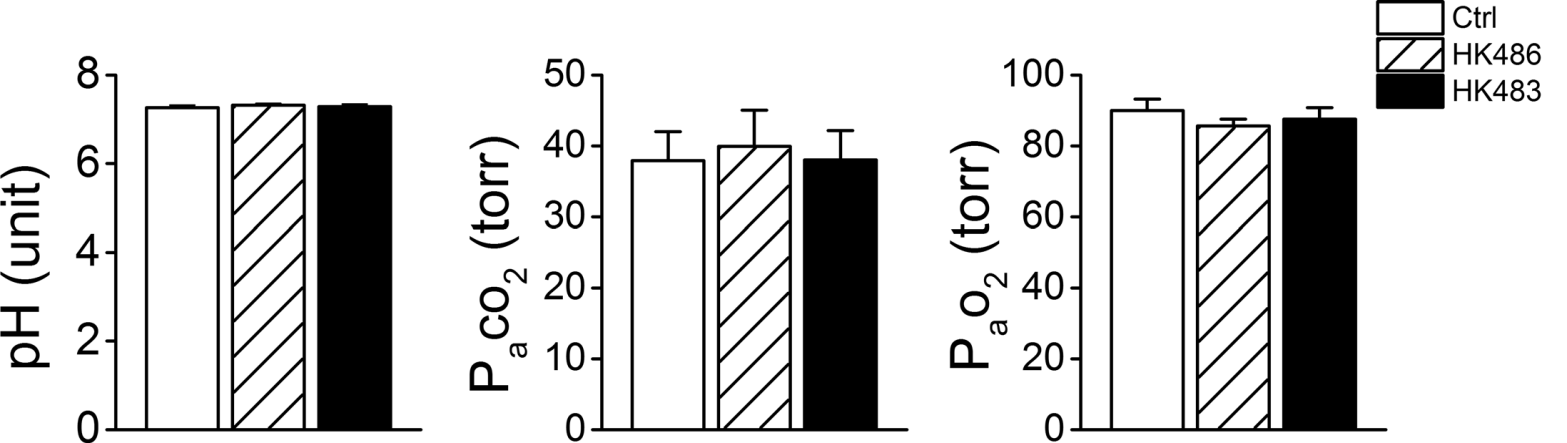
Effects of viral infection on pH and blood gases. Arterial blood was sampled at 3 dpi in anesthetized Ctrl, HK486 and HK483 mice (n = 7/each group). Mean ± SE. There is no difference in blood gases/pH among the three groups at 3 dpi.

We compared arterial blood gases/pH at 3 dpi and found no significant difference among the three groups (Fig 7). It is possible that the tachypnea and reduced breathing variation masked blood gases/pH changes. Anyway, the occurrence of HVR and HCVR depression observed in HK483 mice at 2 and 3 dpi was not due to baseline change in blood gases and pH. Additionally, at 3 dpi, the virus titer (log PFU/g ± SD) in the lungs of HK483 and HK486 mice was similar: 7.1 ± 0.5 and 6.8 ± 0.6. Viremia was not detected in both infected groups (Table 1). These findings suggest that the respiratory ventilation changes observed in HK483 are not the result of changes in lung viral load in this experimental system.

**Table 1:** Virus titer in the blood and lungs at 3 dpi (log_10_ pfu/g, mean ± SD).

Group	Blood	Lungs
Ctrl	-	-
HK486	-	7.1 ± 0.5
HK483	-	6.8 ± 0.6

"-": not detectable. n = 7.

### HK483 virus induces virus replication and up-regulated SP expression in vagal sensory neurons

**Fig 8:**
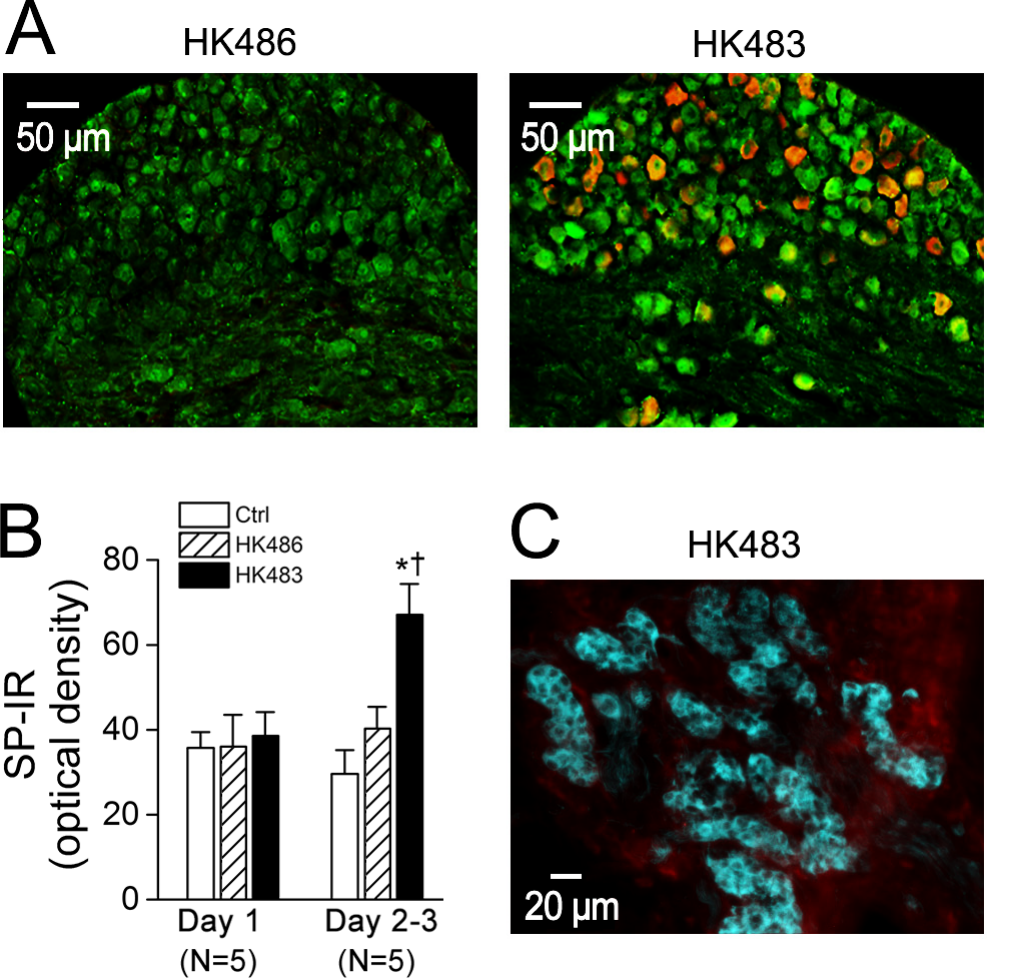
Expression of nucleoprotein-immunoreactivity (NP-IR, orange) in substance P immunoreactivity (SP-IR) neurons (green) in the nodose ganglion and tyrosine hydroxylase immunoreactivity (TH-IR) neurons (cyan) in the carotid body. A: At 2 dpi, nodose ganglion NP-IR appears in a HK483 (right) but not HK486 mice (left) and HK483 virus increases the density of SP-IR in the nodose ganglionic neurons. In contrast, NP-IR is undetectable in the nodose ganglion of infected HK483 and HK486 mice at 1 dpi (data not shown). B: The density of SP-IR in the nodose ganglion is not significantly changed by HK483 virus until 2 dpi. Because of the similarity of the SP-IR data at 2 and 3 dpi (N = 3 and 2, respectively) in either HK483 (64 ± 11 vs. 69 ± 15) and HK486 mice (40 ± 9 vs. 41 ± 11), they were grouped. C: No viral replication in the carotid body of a HK483 mouse in which glomus cells are marked by TH-IR (cyan) at 3 dpi in a HK483 mice. N = 5 for each group; * and † P < 0.05 compared HK483 data to Ctrl and HK486 data respectively.

Considering the important role the carotid body and PCFs play in control of HVR and HCVR, we tested whether lethal H5N1 virus was able to infect both the carotid body and PCFs. NP-IR was utilized to detect virus replication, while co-labeled SP-IR or TH-IR was applied to determine specific virus replication in vagal sensory neurons (C-neurons) or glomus cells in the carotid body, respectively. As the results show, NP-IR was undetectable in the nodose ganglia of HK486 mice, but began to appear in the vagal nodose ganglionic SP-IR neurons of HK483 mice at 2 dpi (Fig 8A and 8B). Interestingly, all NP-IR was co-expressed with SP-IR neurons, indicating a selective virus replication in vagal C-neurons (but not other types of neurons). In addition, HK483 infection failed to markedly elevate SP-IR density in the nodose ganglion until 2 dpi (Fig 8B), implying a stimulating effect of this strain of virus on these vagal sensory neurons to synthesize SP. Surprisingly, NP-IR was not denoted over the early stage of infection in the carotid body of HK483 or HK486 mice (Fig 8C).

## Discussion

Previous studies have shown that intranasal inoculation of lethal H5N1 viruses in mice [7,8], ferrets [9], and cats [10] induces pulmonary infection and infiltration [2,7,33,34] followed by death at 6–8 dpi [2,7,8,35,36]. However, the impact of lethal viral infection on breathing has not been investigated. One of the novel findings of this study is that HK483 but not HK486 virus induces tachypnea and greatly reduced breathing variation at 2 and 3 dpi. Normal variability of breath duration reflects an optimal breathing control ranging from an absence of any variability to a clearly periodic breathing pattern [26] as the result of excitatory and inhibitory inputs from many neural feedback loops at different states. Both respiratory frequency and breathing variation are under neuronal control. This information, along with the lack of changes in body weight and temperature, ${\dot{\text{V}}}_{{\text{CO}}_{2}}$ and arterial blood gases at 2 and 3 dpi of HK483 virus, implies that NK483 virus infects the nervous system at the early stage to induce these respiratory abnormalities. The lack of a cough in our study is similar to other reports in which lethal H5N1 virus did not evoke coughing in ferrets and cats [9,10], likely due to the weaker or lack of cough reflex in these animal species as compared to humans.

The most important finding in the present study is that HK483 viral infection induces significant depression of HVR and HCVR at 2 and 3 dpi. In addition, HK483 virus induces these respiratory disorders without changes in body weight and temperature, ${\dot{\text{V}}}_{{\text{CO}}_{2}}$, and blood gases/pH and without a difference in virus titers of the lungs as compared to HK486 virus. The lack of significant difference of virus titers in the lungs between the two virus strains at 3 dpi is consistent with our previous report [7]. On the other hand, viral titers in the lungs were reportedly higher in HK483 than HK486 mice at 1 or 2 dpi [7,37], which raises a concern as to if this difference facilitates replication of HK483 virus in vagal C-neurons to precede the changes in HVR and HCVR. However, this assumption is not supported by our recent study (unpublished observation by Zhuang and Xu) showing the failure to detect HK486 viral replication in the vagal C-neurons 6 dpi (3 days after HK486 viral infection reaching the same level of HK483 viral titers in the lungs). Our findings suggest blunted ventilatory chemoreflexes by H5N1 viral infection in mice, which again supports the notion that lethal H5N1 viral infection is able to impact the nervous system at the early stage of infection. Given that HVR and HCVR depression occurred during the course of influenza, we further analyzed the correlation between the severity of these disorders and the death date in these animals. Interestingly, HK483 mice, who presented more severe depression of HVR and HCVR at 2 and 3 dpi, died earlier. This close correlation points to a possible contribution of HVR/HCVR depression at the early stage of lethal H5N1 viral infection to developing respiratory failure and death by lethal H5N1 viral infection (see Fig 6B and 6C). In fact, influenza viral infections by other strains, such as H1N1 and H3N2, could also develop hypoxemia, neurological symptoms with brain infection or lesion [38,39], and respiratory failure in some patients [38–41]. It is interesting to determine in future whether infection with H1N1 or H3N2 influenza virus at early stage is able to cause similar HVR and HCVR depression.

In our subsequent experiments, we probed the infection of lethal H5N1 virus on the carotid body and PCFs, two key peripheral nerve systems in control of breathing. We found absence of viremia and virus replication in the carotid body, which is not supportive of the carotid body involvement. Surprisingly, HK483 virus affected vagal sensory neurons at 2 and 3 dpi with following characteristics. First, NP-IR was undetectable in the nodose ganglia of HK486 mice, but abundantly appeared in the vagal nodose ganglionic SP-IR neurons of HK483 mice. In agreement, the neurotropic nature of lethal H5N1 virus has been reported in peripheral nerves (Auerbach’s plexus of the enteric nervous system) and the CNS [7,8] in mice. Second, all NP-IR was co-expressed with SP-IR neurons. In other words, NP-IR was only expressed in vagal sensory C neurons marked by SP-IR without NP-IR expression outside of these neurons in the nodose of ganglion. Considering that H5N1 virus is intranasally inoculated and its titers were found in lungs, some of co-labeled neurons in the nodose ganglion (Fig 8A), at least in part, are vagal pulmonary C-neurons (PCFs). Consistent with our finding, HSV-1 is reported to replicate faster in C- (<36 h) than Aσ-fibers (>66 h) [42]. These data suggest that HK483 but not HK486 virus is neurotropic and first infects vagal C-neurons, rather than Aσ-neurons, at the early stage of infection. Third, HK483 but not HK486 viral infection profoundly elevated SP-IR density in the nodose ganglion, suggesting a stimulating effect of this strain virus on vagal sensory neurons and overexpression of C-fibers (plasticity). Taken together, our morphological data lead to the conclusion that lethal H5N1 viral infection at the early stage is able to infect and stimulate vagal (pulmonary) sensory neurons without viremia and viral replication in the carotid body. Because of the absence of NP-IR in the brainstem 2 and 3 days after lethal H5N1 viral infection in mice [7,8], we do not expect that NP-IR would be expressed in the brainstem at 2 and 3 dpi in our HK483 mice.

There are two lines of evidence from this study pointing to PCFs’ involvement in the respiratory disorders noted at 2 and 3 dpi. We found that tachypnea and reduction of breathing variation uniquely occurred in HK483 but not HK486 mice. These respiratory disorders are independent of varied pulmonary inflammation because our previous study has shown a similar pulmonary inflammation induced in HK483 and HK486 mice [7]. Tachypnea is thought to be induced by activation of PCFs under the pulmonary disorders (see reviews [43,44]), thus, our data support PCF involvement in the tachypnea and reduction of breathing variation. We also found that HK483 viral infection markedly increased SP-IR expression of vagal C-neurons at 2 dpi temporally coincident with appearance of respiratory disorders (tachypnea, lowered respiratory variation, depressed HVR and HCVR), supporting vagal C-fiber activation and/or sensitization. PCF activation could depress HVR [20,21,45] and CO_2_ chemoreception of the retrotrapezoid nucleus [22] in the animals. Therefore, our data suggest that lethal H5N1 virus infects and stimulates vagal pulmonary C-neurons (PCFs) likely to contribute to the respiratory disorders denoted at 2 and 3 dpi.

In summary, HK483 but not HK486 virus induces respiratory disorders including tachypnea, reduction of breathing variation, and decreases in HVR/HCVR at 2 and 3 dpi concomitant with viral replication and upregulated SP expression in vagal sensory neurons. Given that PCFs are responsible for control of respiratory frequency and inhibition of HVR and HCVR, the lethal H5N1 viral infection-induced respiratory disorders in this study may result from PCF sensitization and/or activation by the lethal viral replication.

## Perspective

Patients infected by lethal H5N1 virus initially present cough, dyspnea, pulmonary inflammation, and infiltration and subsequently show respiratory failure and death several days later, primarily due to respiratory failure [3–6,46–51]. Owing to the lack of an effective vaccine, it is important to develop corresponding effective treatments applied at the early stage of infection to prevent the subsequent respiratory failure. However, it has not been explored if the respiratory disorders occur at the early stage of the lethal viral infection and to what extent the disorders are correlated to the respiratory failure (death). Our results reveal, for the first time, the presence of respiratory disorders (tachypnea, reduction of breathing variation, and decreases in HVR/HCVR) at the early stage of HK483 virus and the close relationship between depression of HVR/HCVR and the death, which is clinically relevant and has a three-folded significance. Our results of HVR/HCVR depression observed at 2 and 3 dpi in HK483 mice may provide a potential predictor for the lethality of H5N1 viral infection at the early stage of infection in the clinical setting. Their correlation to the death gain insight into the mechanisms underlying the development of the respiratory failure. More importantly, confirmation of these respiratory disorders in our HK483 mice may be beneficial to developing therapeutic intervention with respiratory failure after the lethality of H5N1 viral infection. Lethal H5N1 viruses are known to be neurotropic with replication in peripheral afferents 3 dpi and the CNS thereafter in mice [8]. Our finding that HK483 virus replicates and up-regulates SP expression in vagal sensory neurons at 2 and 3 pdi not only extends the existing knowledge of neurovirology of the H5N1 virus, but also provides morphological evidence of vagal pulmonary neural plasticity (activation) induced uniquely by lethal H5N1 virus. Further studies are necessary to determine i) whether lethal H5N1 virus induces PCF sensitization/activation, and if so how; ii) if PCF sensitization/activation is causal to the lethal H5N1 virus-induced respiratory disorders and failure; and iii) what potential treatments applied at the early stage of lethal viral infection are able to prevent the respiratory disorders and minimize the mortality.
